# 3D visualization of extracellular vesicle uptake by endothelial cells

**DOI:** 10.1186/s11658-018-0123-z

**Published:** 2018-12-17

**Authors:** Martyna Durak-Kozica, Zbigniew Baster, Karol Kubat, Ewa Stępień

**Affiliations:** 1 Department of Medical Physics, Institute of Physics, Jagiellonian University, Lojasiewicza 11, Kraków, Poland; 2 Department of Molecular and Interfacial Biophysics, Institute of Physics, Jagiellonian University, Lojasiewicza 11, Kraków, Poland

**Keywords:** Extracellular vesicles, Internalization, Confocal microscopy, Endothelial cells, 3D visualization

## Abstract

**Background:**

Extracellular vesicles are small vesicles that contain cytoplasmic and membrane components from their paternal cells. They enter target cells through uptake to transfer their biological cargo. In this study, we investigated the process of endothelial EV internalization and created a 3D visualization of their intracellular distribution.

**Methods and results:**

Two immortalized endothelial cell lines that express h-TERT (human telomerase) were used for EV release: microvascular TIME and macrovascular HUVEC. EVs were isolated from the cell culture medium via differential centrifugation and used for the uptake experiments. The size distribution of the EVs was measured using TRPS technology on a qNano instrument. Internalization of EVs was observed using a Zeiss LSM 710 confocal laser microscope after staining of the EVs with PKH26. EVs were observed intracellularly and distributed in the perinuclear region of the target cells. The distribution patterns were similar in both cell lines.

**Conclusion:**

The perinuclear localization of the internalized EVs shows their biological stability after their uptake to the endothelial cells. The 3D visualization allows the determination of a more accurate location of EVs relative to the donor cell nucleus.

**Electronic supplementary material:**

The online version of this article (10.1186/s11658-018-0123-z) contains supplementary material, which is available to authorized users.

## Introduction

Extracellular vesicles (EVs) are nanosized, membrane-derived vesicles. Based on their sizes and biological properties, they are divided into three groups: *exosomes*, which range between 50 and 100 nm; *ectosomes*, which range between 100 and 1000 nm in diameter; and *apoptotic bodies*, which are over 1000 nm in diameter [1].

EVs also vary in the way they are produced and released. Exosomes originate from multi-vesicular bodies (MVBs), whereas ectosomes are released from the cell membrane in a shedding process. The formation of apoptotic bodies takes place at the end of the apoptosis process [2].

Several experimental studies have shown that EVs contain various proteins, bioactive lipids, miRNAs and even mRNAs, and that they transfer them between cells contributing to cell-to-cell communication [3–7]. EVs might be internalized by cells in a variety of endocytic pathways (e.g., clathrin-dependent endocytosis [8, 9]) and clathrin-independent pathways (e.g., macropinocytosis [10–12], phagocytosis [10, 13], caveolin-mediated uptake [10, 14–16], lipid raft-mediated internalization [17–19]). The glycoproteins (e.g., HSPG [20]) and proteins (e.g., tetraspanins [21–24], integrins [25, 26]) on the surfaces of EVs and their target cells are known to determine the uptake mechanism. However, the precise molecular uptake mechanisms and cellular fate of EVs are still unknown. For example, it is not known how they are taken up by endothelial cells. Clathrin-independent endocytosis with some contribution of lipid transfer seems to be most likely [27, 28].

Endothelial cells are vascular cells with paracrine and autocrine properties. By secreting EVs, they contribute to both coagulation and fibrinolysis. They also respond to different pro- and anti-proinflammatory signals [6]. After internalization, endothelial-derived exosomes have beneficial or detrimental effects on the targeted endothelial cells by improving their angiogenic properties or maintaining a pathogenic phenotype [7, 29].

The aim of our study was to evaluate whether endothelial-derived EVs might be taken up by endothelial cells and to assess whether they can act as paracrine factors for neighboring cells in further studies. We also wanted to show the intracellular distribution of endothelial-derived EVs in the targeted endothelial cells to gain a better insight into EV trafficking mechanisms. The proposed approach should be suitable to investigate EV fate in further experiments.

## Material and methods

### Materials

The immortalized hTERT cell lines telomerase immortalized human microvascular endothelium (TIME; CRL-4025) and human umbilical vascular endothelial cells (HUVEC; CRL-4053) were purchased from LGC Standard. Vascular cell basal medium (ATCC PCS-100-030) and supplements were purchase from LGC Standard. Antibiotics and exosome-depleted fetal bovine serum (FBS) were purchased from Gibco (Thermo Fisher Scientific; A2720801). Bovine serum albumin (BSA) and red fluorescent PKH26 dye (PKH26GL) for EV staining were purchased from Sigma-Aldrich. For the endothelial cell culture, 75-cm^2^ bottles were used. For confocal microscopy observations, BIO-PORT glass bottom dishes (thickness #1.5) were purchased from Cellvis.

### Cell culture

TIME cells were cultured in vascular cell basal medium supplemented with penicillin (100 U/ml), streptomycin (100 U/ml), blasticidin (12.5 μg/ml) and Microvascular Endothelial Cell Growth Kit-VEGF (ATCC PCS-110-041). HUVECs were cultured in vascular cell basal medium supplemented with penicillin (100 U/ml), streptomycin (100 U/ml), and Endothelial Cell Growth Kit-VEGF (ATCC PCS-100-041). All cells were cultured at 37 °C with 5% CO_2_.

### Isolation of EVs

**Fig. 1:**
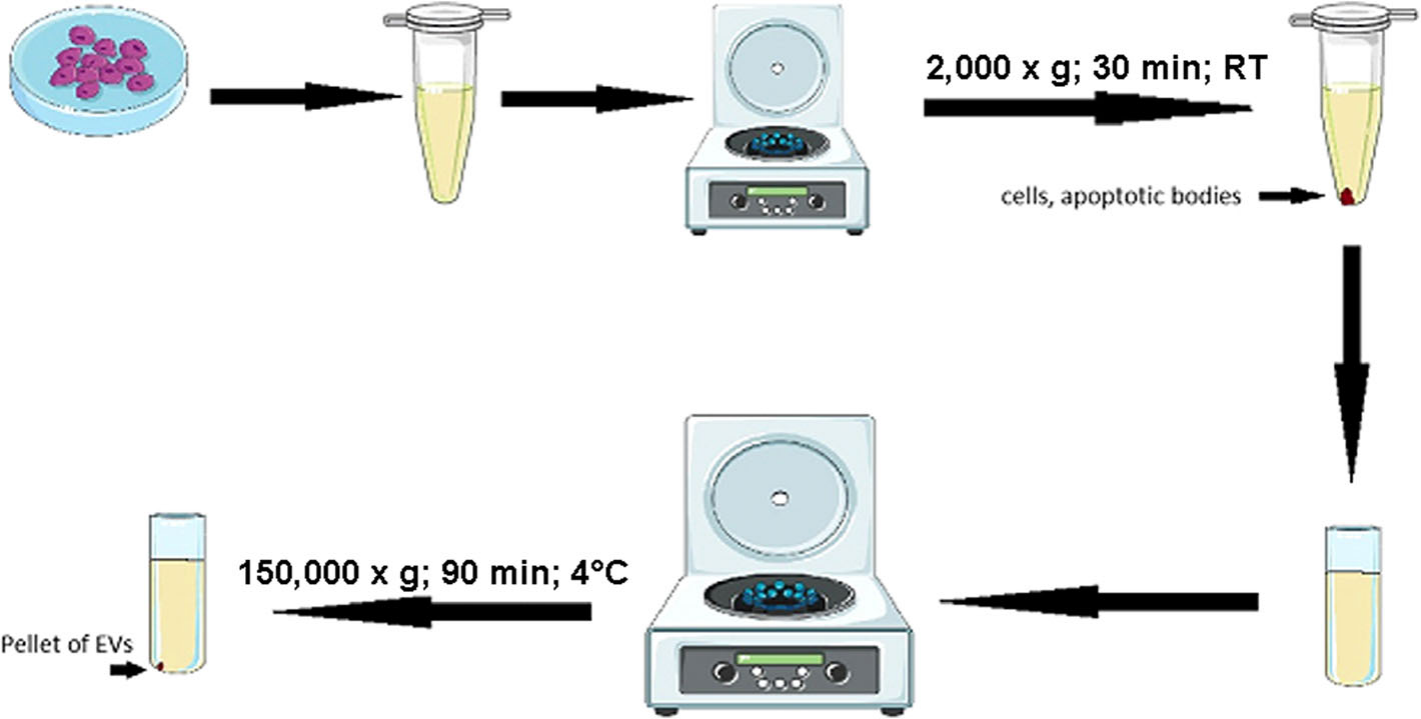
The workflow for EV isolation. Adapted from [39]

Endothelial cells were seeded on cell culture dishes to obtain 85% confluence. For EV isolation, TIME cells and HUVECs were cultured for 48 h with 2% exosome-depleted FBS. After that, cell culture media were harvested and centrifuged at 2000 x g for 30 min at room temperature to remove cells and apoptotic bodies. Supernatants were collected and ultracentrifuged for 90 min at 150,000 x g and 4 °C to obtain the EV pellet. A schematic description of the procedure is presented in Fig. 1. The obtained EV pellets were diluted in PBS or culture medium for qNano or internalization measurements, respectively.

### EVs size analysis

The size distribution and concentration of EVs were measured with tunable resistive pulse sensing (tRPS) technology using qNano system (Izon Science Ltd.). The instrument was set up and calibrated using CPC100 beads (Izon Science) according to the manufacturer’s instructions. EV samples were diluted 3 times in PBS (Sigma). The EVs were measured using a NP100 nanopore (analysis range 50–330 nm; Izon Science) with 10 mbar pressure. Voltage and stretch were set to give a stable current between 100 and 120 nA. Samples were analyzed for 3 min or until 1000 vesicles were counted. Data processing and analysis were done on the Izon Control Suite software v2.2. The Gauss distribution was matched to histograms.

### Cellular uptake of endothelial-derived EVs

Endothelial-derived EVs were labelled with PKH26 as previously described with a minor modification [30]. In brief, 0.1 μl of PKH26 was added to a pellet of EVs in a total of 50 μl of diluent C and incubated for 20 min at room temperature. A sample without EVs was used as a negative control to determine any carryover of PKH26 dye. Then, EVs were blocked with 50 μl of 1% BSA, dissolved in 900 μl of phosphate buffered saline (PBS) and ultracentrifuged under the same conditions. After this step, the supernatant was discarded and the pellet of EVs was washed in 1 ml of PBS and ultracentrifuged once more. The pellet containing PKH26-labeled EVs was resuspended in 1 ml of cell culture medium.

For confocal examinations, TIME cells and HUVECs were cultured in glass bottom dishes to reach 50% confluence. A medium containing EVs was added for 24 h of incubation. The cultures were then washed 3 times with PBS and fixed with cold (− 20 °C) acetone for 5 min at − 20 °C. DAPI staining was used to visualize nuclei. Cellular uptake of endothelial extracellular vesicles was observed and recorded using Zeiss LSM 710 confocal laser microscope with an oil-immersion Plan-Apochromat 40x NA 1.4 objective (Carl Zeiss Microscopy GmbH), and lasers 405 nm (DAPI) and 561 nm (Pkh26). Images were collected with a voxel size of 0.209 × 0.209 × 0.436 μm, in two lateral and axial directions, respectively, with the ae range set to cover a whole cell in a single image (9.16–23.11 μm).

### Data visualization

The internalized EVs were stained with a lipophilic PKH26 dye. The microscopy 3D data reconstruction was made using a maximum intensity projection algorithm implemented in the Zeiss ZEN lite blue 2.5 software (Carl Zeiss Microscopy GmbH). This 3D visualization method is based on the projection of the most intense voxels along rays orthogonal to the projection plane. A sequence of projections from different adjacent points of view of the sample was made afterwards to create a rotating visualization. Using this method, we could present the localization of EVs inside the cell relative to other structures, such as the nucleus.

## Results and discussion

**Fig. 2:**
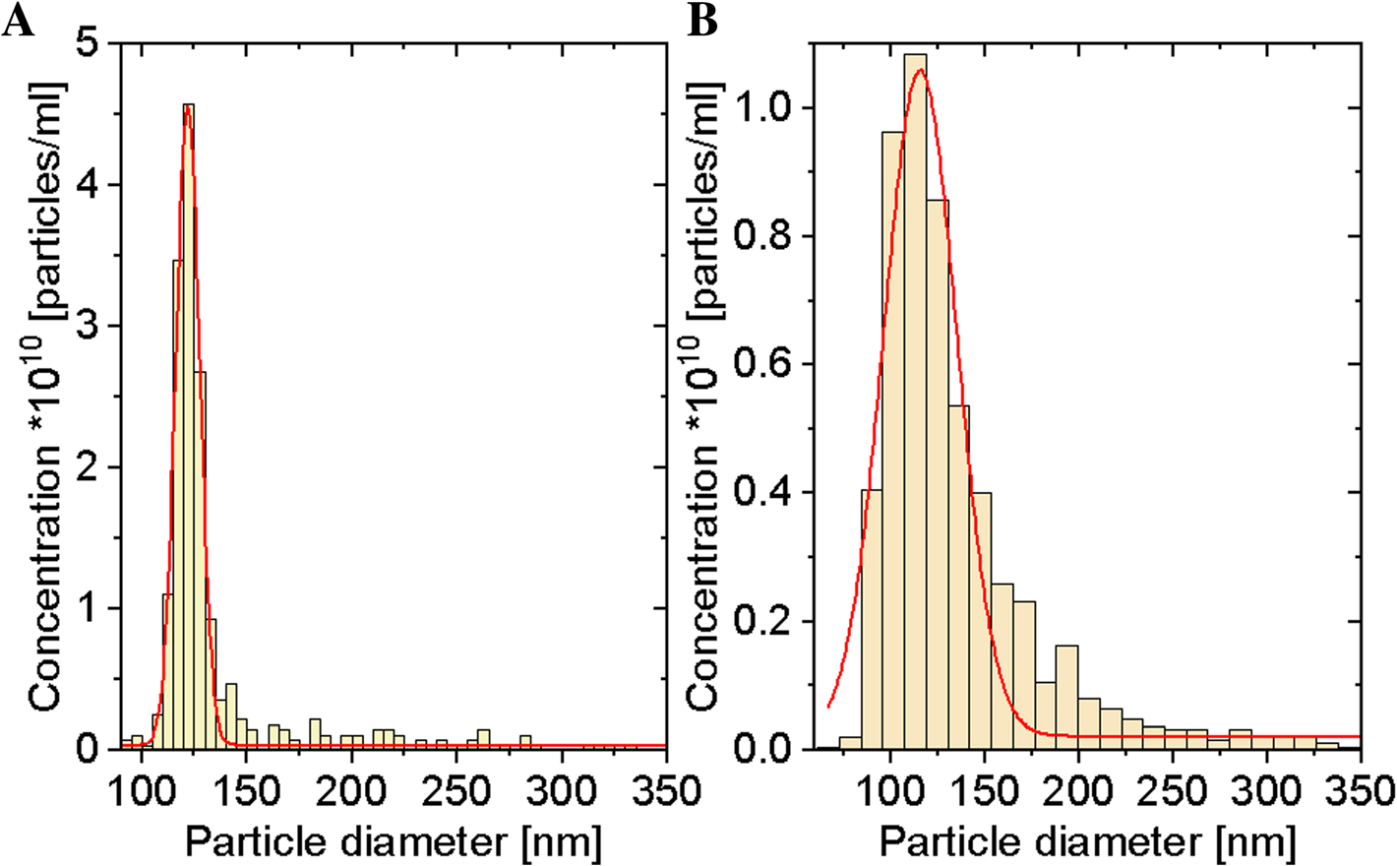
Size distribution of EVs derived from the TIME cell line (**a**) and the HUVEC line (**b**)

qNano measurements **(**Fig. 2a, b**)** revealed that median size of EVs collected from the TIME cell line culture was 121.84 ± 0.08 nm and from the HUVEC line was 115.82 ± 0.96 nm. These results demonstrated that our EV samples included exosomes and ectosomes.

**Fig. 3:**
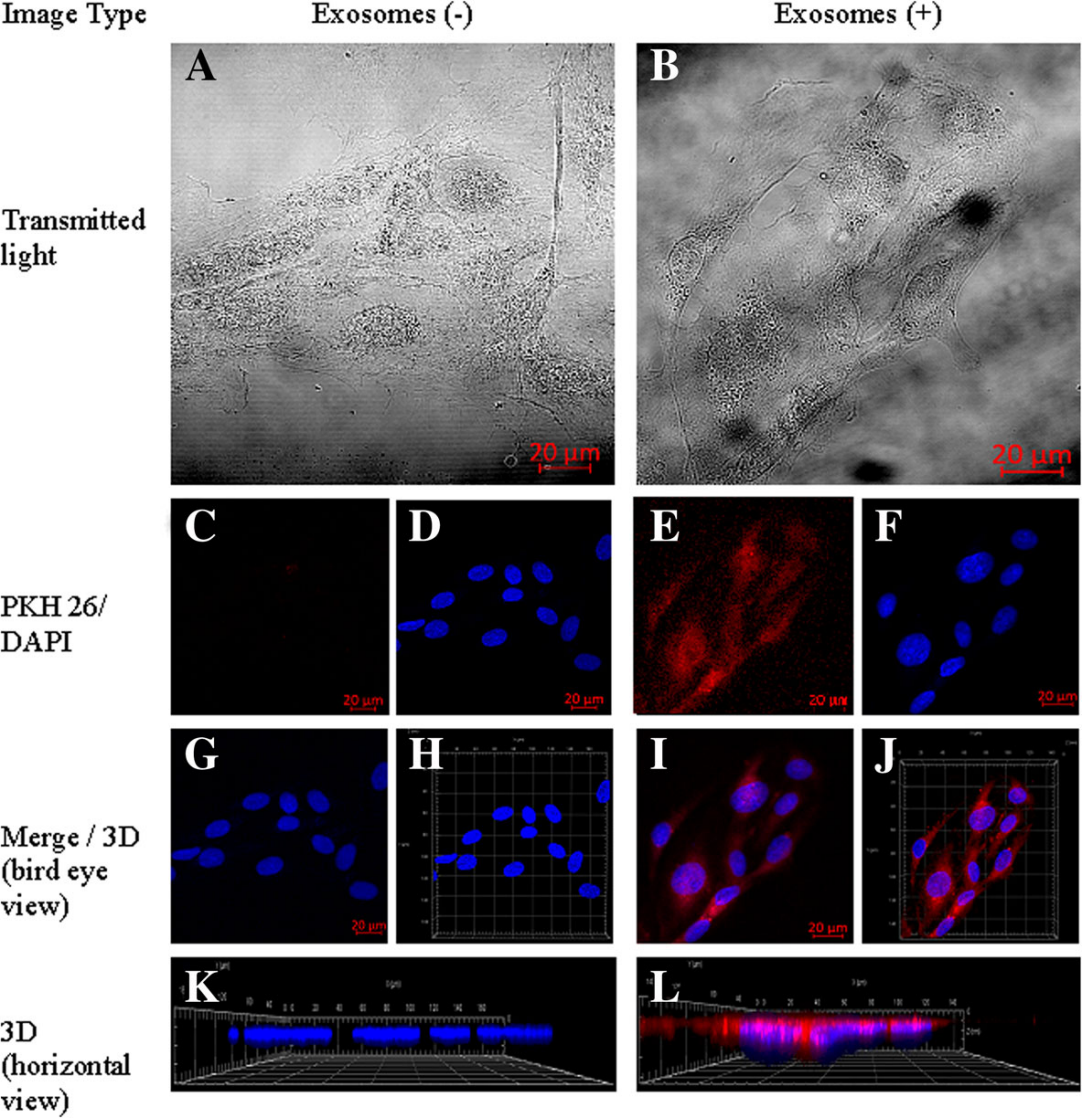
Cellular internalization of HUVEC-derived EVs into HUVECs. HUVECs were incubated for 24 h with EVs labelled with PKH26 (red). The carryover of PKH26 was observed when cells were incubated with PKH26 without EVs (negative control). **a**, **b** – Transmitted light. **c**, **e** – PKH26 staining. **d**, **f** – DAPI staining. **g**, **i** – Merged 2D view. **h**, **j** – Merged 3D view. **k**, **l** – 3D horizontal view

**Fig. 4:**
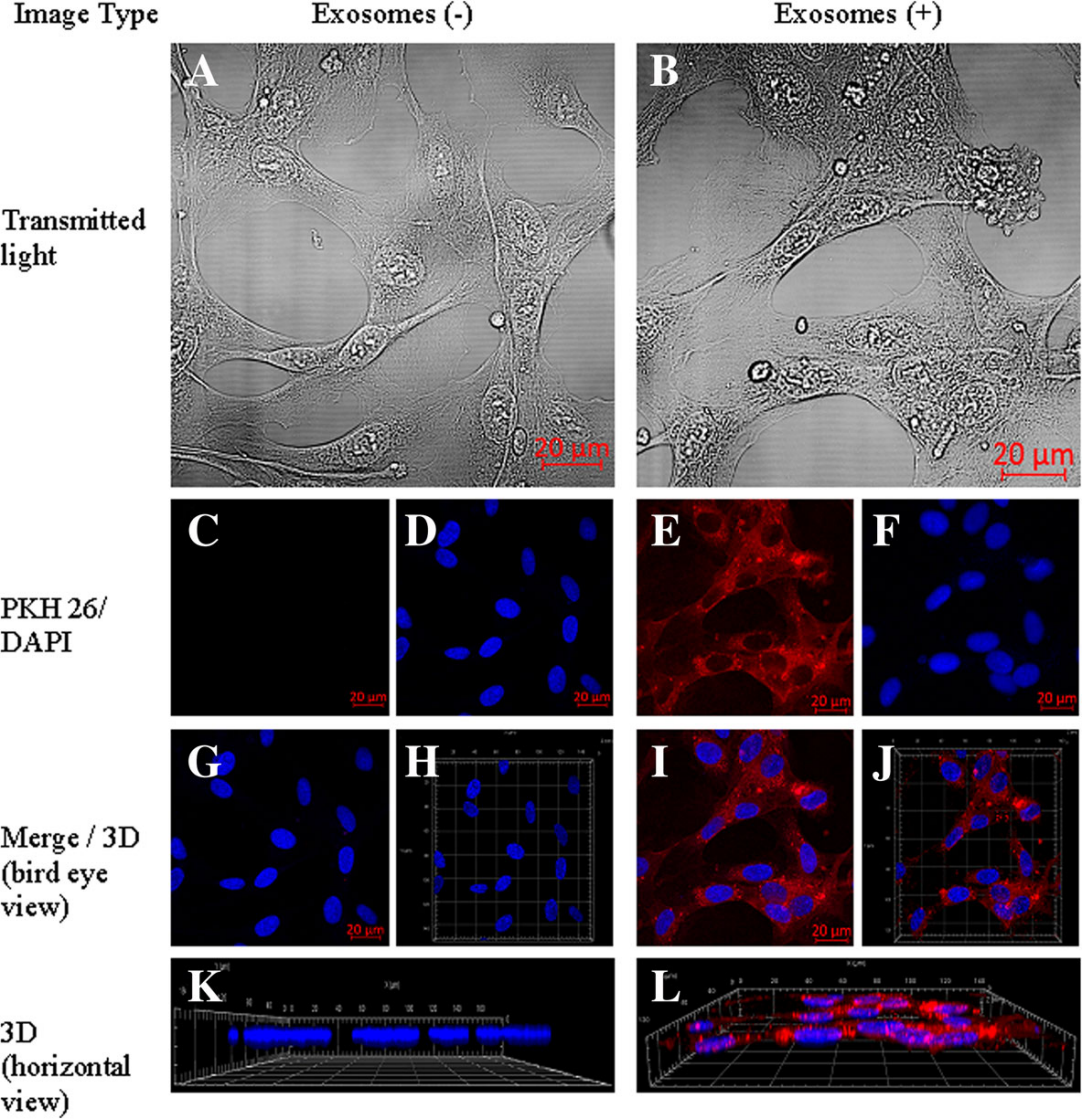
Cellular internalization of TIME-derived EVs into TIME cells. TIME cells were incubated for 24 h with EVs labelled with PKH26 (red). The carryover of PKH26 was observed when cells were incubated with PKH26 without EVs (negative control). **a**, **b** – Transmitted light. **c**, **e** – PKH26 staining. **d**, **f** – DAPI staining. **g**, **i** – Merged 2D view. **h**, **j** – Merged 3D view. **k**, **l** – 3D horizontal view

We observed that PKH26-labeled EVs, after internalization, were localized in the cytoplasm of both macrovascular (HUVEC) and microvascular (TIME) cells. This suggests that EVs can be internalized by different types of endothelial cell (Figs. 3 and 4). Endothelial cells can take up lipid-rich vesicles (e.g., LDL, EVs) and accumulate them for a long time [27, 28]. It has been suggested that lipid components, like phosphatidylserine (PS) or cholesterol, have the most important role in EV uptake by the endothelium. This hypothesis has been proven using inhibition of EV internalization by annexinV-PS binding and using cholesterol synthesis breakout [27, 28]. Interestingly, lactadherin blocking by lactadherin antibodies also disturbs EV internalization [31]. Lactadherin is a PS-binding membrane protein with Ca-independent activity [32]. That suggests a very complex mechanism of EV internalization involving a number of cellular pathways. Under control conditions, no PKH26 dye uptake was observed.

Our setup allowed us to achieve a lateral resolution of 160 nm and an axial resolution 608 nm. To reduce scanning time, we reduced our sampling step in the lateral direction. As per the Nyquist-Shannon sampling theorem, this resulted in an actual resolution of 218 nm and 872 nm in the lateral and axial dimensions, respectively. It was still possible to distinguish regions of interest inside the imaged cells.

Our 3D visualization of internalized EVs allowed us to assess the localization of EVs mostly in the perinuclear region. These results concur with results obtained by Mantel et al. [33], who observed perinuclear localization of RBC-derived EVs in bone marrow endothelial cells (BMEC). Lombardo et al. [34] also observed internalization of endothelial-derived EVs by endothelial cells.

We also showed that EVs did not remain attached to the outer cell membrane, but all penetrated into the cytoplasm (Additional file 1: Movie 1, Additional file 2: Movie 2, Additional file 3: Movie 3, Additional file 4: Movie 4). As we showed here, it is clear that there are two distinct regions within the cell corresponding to the nuclear and microvesicular regions. Moreover, we showed that in some cases, the nuclei can be positioned over microvesicles, which admittedly shows an intracellular location of microvesicles.

The 3D data reconstruction allowed us to demonstrate EV internalization and intracellular localization (Additional file 1: Movie 1, Additional file 2: Movie 2, Additional file 3: Movie 3, Additional file 4: Movie 4). To the best of our knowledge, such a technique had not previously been used for a presentation of 3D EV uptake in endothelial cells. Note that the intracellular EV localization can also be distinguished after treatment of the cell surface with trypsin [35].

Previously, EV internalization has been observed by means of confocal microscopy after staining with different fluorescent lipid membrane dyes including rhodamine B [5, 6], DiD [36], DiI [36] and PKH26 [7, 30]. Lipophilic PHK26 dye has also been used for visualization of EV uptake using imaging flow cytometer methods [36]. Another group of dyes are membrane-permeable chemical compounds, such as carboxyfluorescein succinimidyl ester (CFSE), which binds covalently to intracellular lysine residues and other amine sources in EVs. In such staining, microtubule and EV co-localization has been observed [37].

In comparison to other co-localization microscopy-based methods [36], this method does not require any kind of sophisticated staining. It only needs a stain to define the cell shape (like actin staining, fluorescent protein synthesis, fluorescein diacetate uptake or only partial shape staining, e.g. nucleus staining). Furthermore, in the future, it might be possible to combine this method with the 3D virtual reality visualization methods that have emerged in recent years [38].

That said, we currently find two issues with a such idea. Most computer setups are insufficient to provide live rendering at around 24 frames/s, which is required by the MIP algorithm. This will either cause lack of fluency in visualization or a decrease in projection accuracy and its resolution. Cost may also be an issue. Even though Stefani et al. say that the hardware investment is a small percentage of the cost of a confocal microscope, it is worth pointing out that most groups use shared equipment or must pay for the time they use other groups’ equipment. For analyses and studies of other groups’ publications, more than one workstation is probably needed, which will escalate the costs.

For now, the method presented allows sufficiently deep studies of problems connected with internalization of EVs. Our video presentations should allow researchers to become acquainted with the results that are currently possible.

## Conclusions

Our results indicate that EVs are taken up by micro- and macrovascular endothelial cells. The presented 3D visualizations clearly indicate EV uptake and perinuclear localization. Finally, our approach can be used for further studies on the mechanism of endothelial cell activation after EV exposure.

## Additional files

Additional file 1: **Movie 1.** Visualization of EVs internalization. TIME cells turn around X. (AVI 1715 kb)

Additional file 2: **Movie 2.** Visualization of EVs internalization. TIME cells turn around Y. (AVI 1423 kb)

Additional file 3: **Movie 3.** Visualization of EVs internalization. HUVEC cells turn around X. (AVI 1456 kb)

Additional file 4: **Movie 4.** Visualization of EVs internalization. HUVEC cells turn around Y. (AVI 1644 kb)

## Abbreviations

3D: Three dimensional
BMEC: Brain microvascular endothelial cells
BSA: Bovine serum albumin
CFSE: Carboxyflurescein succinimidyl ester
CPC100: Calibration particles, size 100 nm
DAPI: 4′,6-Diamidino-2-Phenylindole
EV: Extracellular vesicles
FBS: Fetal bovine serum
HSPG: Heparan sulfate proteoglycan
hTERT: Human telomerase reverse transcriptase
HUVEC: Human umbilical vein endothelial cells
miRNA: Micro Ribonucleic acid
mRNA: Messenger Ribonucleic acid
MVB: Multivesicular bodies
NP100: Nanopore, 100 nm
PBS: Phosphate-buffered saline
PS: Phosphatidylserine
RBC: Red blood cells
TIME: Endothelial cells immortalized with hTERT
tRPS: Tunable Resistive Pulse Sensing
